# Supplementary material for: Enzymatic enhancing of triplet–triplet annihilation upconversion by breaking oxygen quenching for background-free biological sensing
Source: Nat Commun. 2021 Mar 26;12:1898. doi: 10.1038/s41467-021-22282-1 (PMC7997900; doi:10.1038/s41467-021-22282-1)
Supplement: Supplementary file 1 — Supplementary Information [file 41467_2021_22282_MOESM1_ESM.docx]

**Supplementary Information**

Enzymatic Enhancing of Triplet-triplet Annihilation Upconversion by Breaking Oxygen Quenching for Background-free Biological Sensing

Ling Huang, Timmy Lee, Kai Huang, Gang Han*

Department of Biochemistry and Molecular Pharmacology, University of Massachusetts Medical School, Worcester, MA 01605, United States.

Email: Gang.Han@umassmed.edu


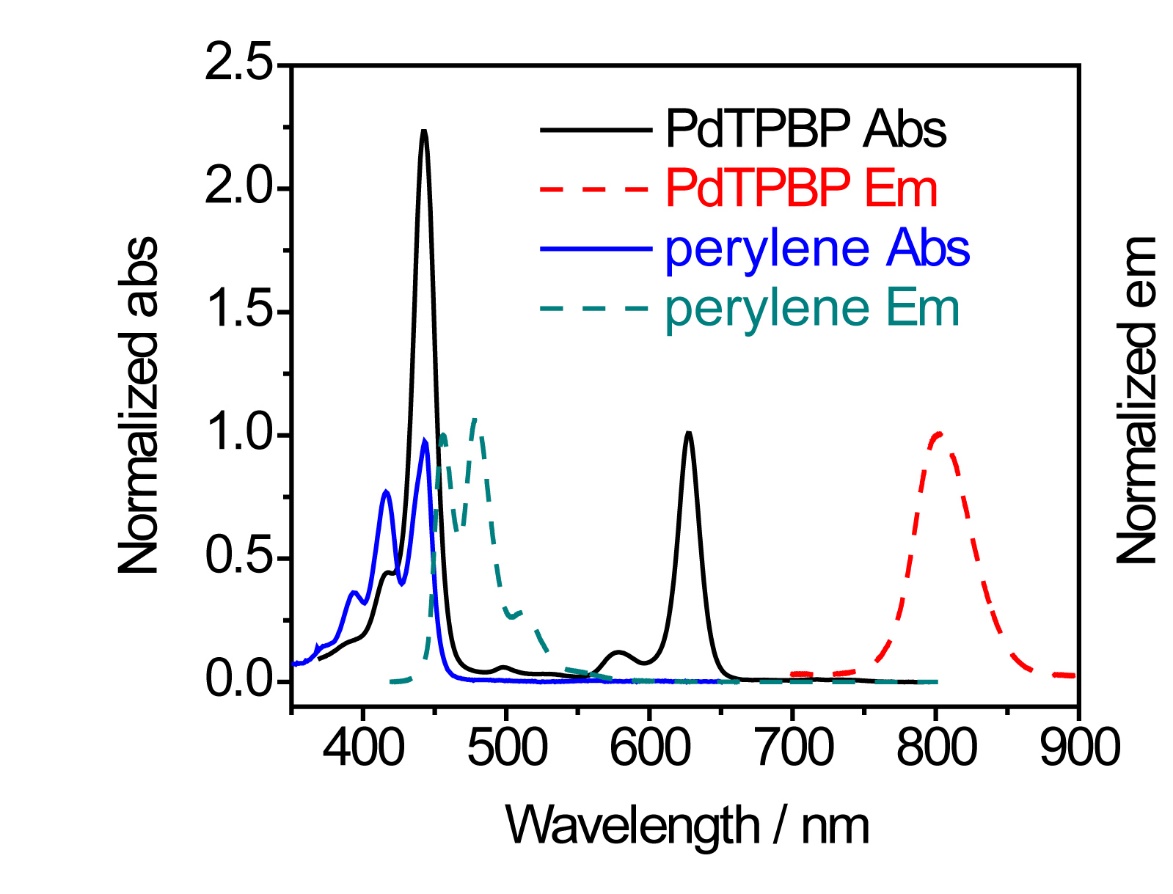

**Supplementary Figure 1**. Normalized absorption and emission spectra of PdTPBP and perylene in degassed toluene.


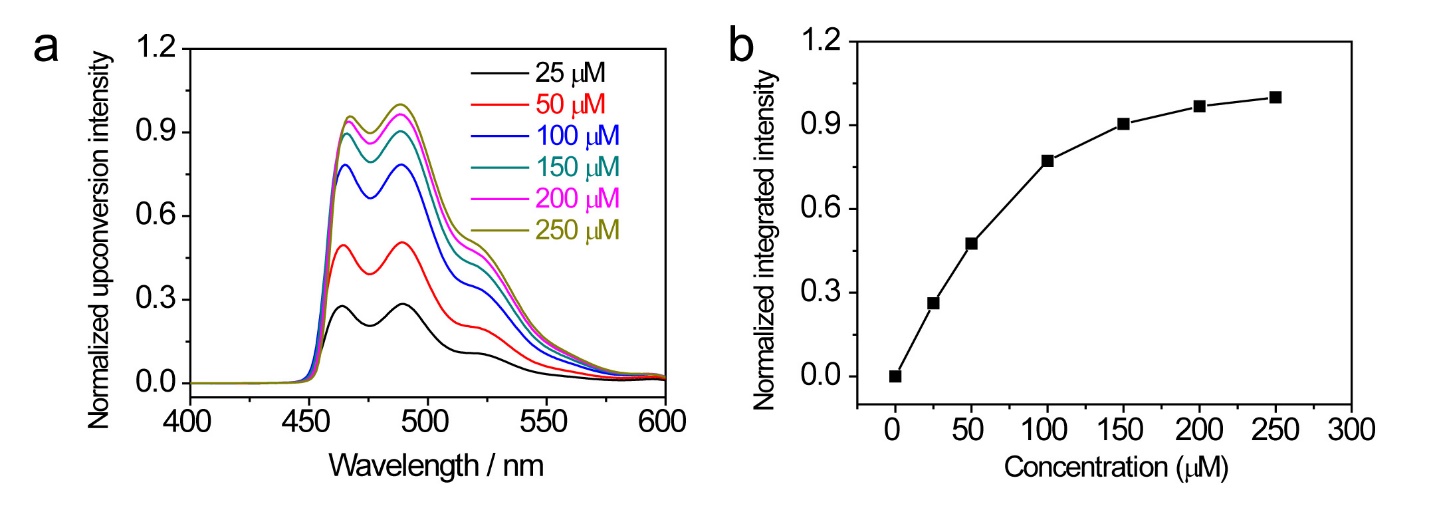


**Supplementary Figure 2**. **The perylene-concentration dependent upconversion.** **a** Upconversion emission spectra of mixed solutions of PdTPBP (10 μM) with different concentrations of perylene in degassed toluene. **b** The respective integrated TTA upconversion intensity as a function of perylene concentration was drawn. Toluene, *λ*_ex_ = 650 nm, 100 mW cm^-2^.


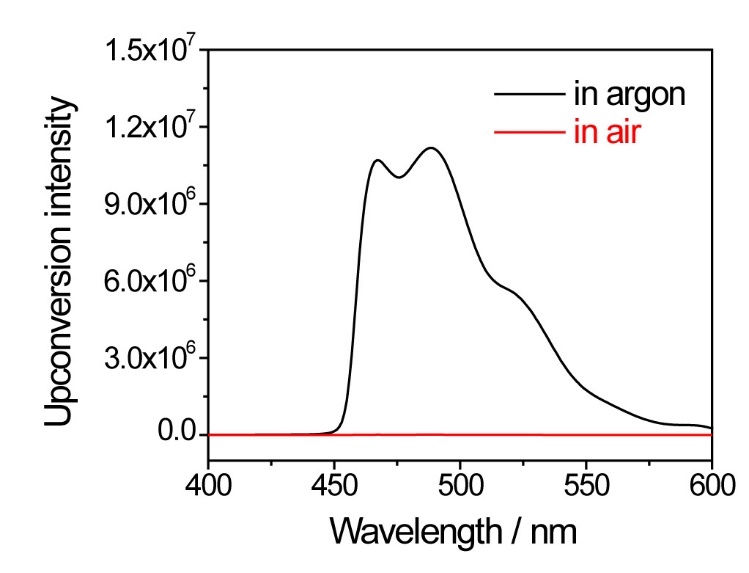


**Supplementary Figure 3**. Upconversion emission spectra of PdTPBP (10 μM) and perylene (250 µM) in argon and air. (solvent: toluene, *λ*_ex_ = 650 nm, 100 mW cm^-2^).


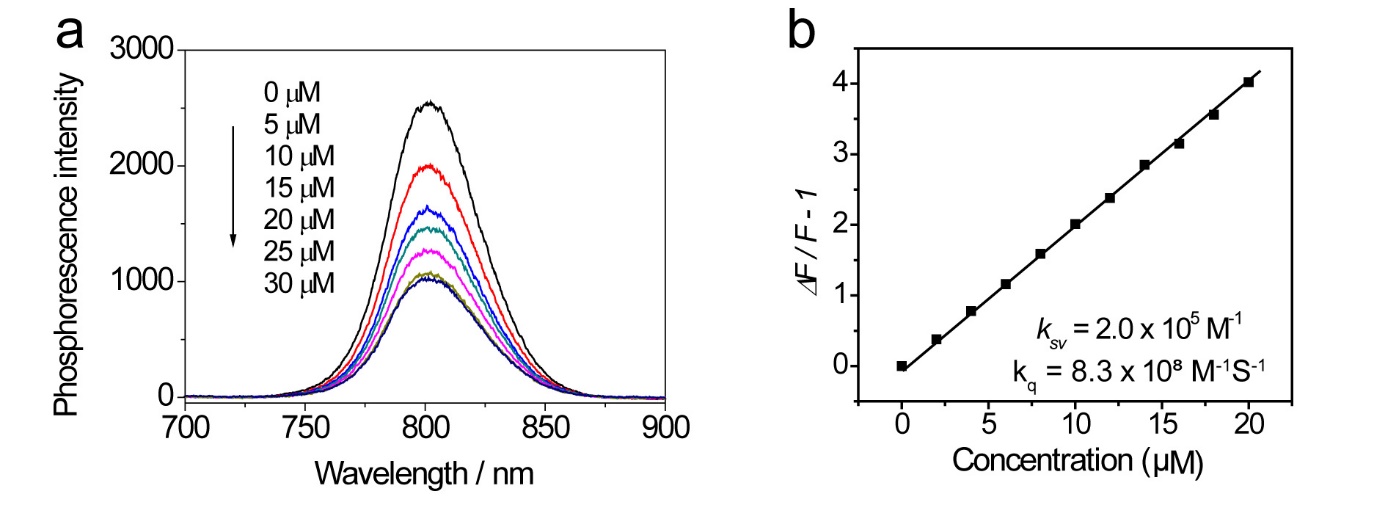


**Supplementary Figure 4**. **Efficient triplet triplet energy transfer from PdTPBP to perylene.** **a** Phosphorescence quenching of PdTPBP (*λ*_ex_ = 635 nm) with the increasing perylene concentration in deaerated toluene. *c* (PdTPBP) = 10 μM. **b** The Stern–Volmer quenching curve of the phosphorescence of PdTPBP in the presence of perylene, in toluene. *k*_q_ = *k*_sv_ / τ_T_, *k*_q_ is bimolecular quenching constant, τ_T_ is the triplet excited lifetime of PdTPBP (τ_T_ = 252 µs).


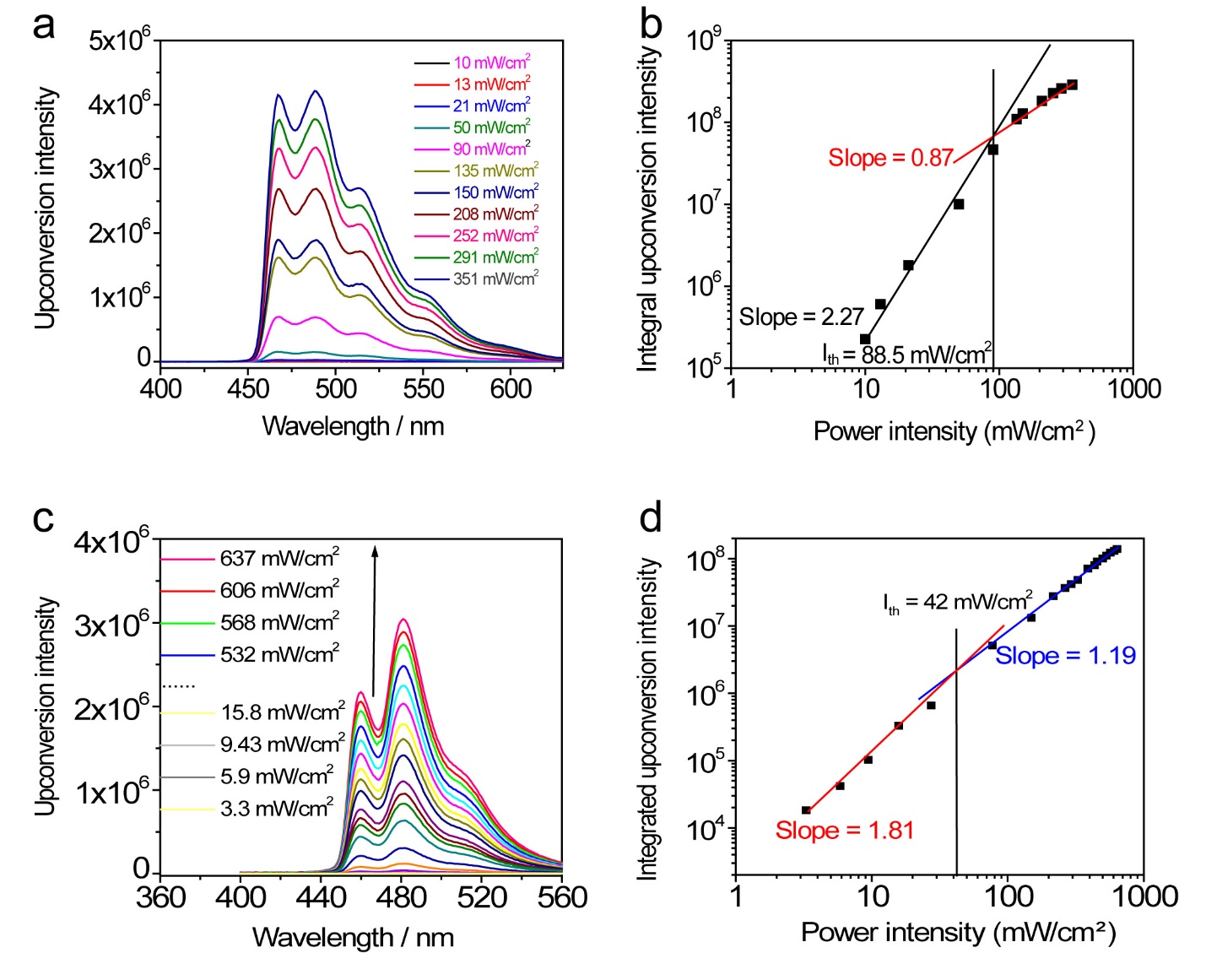


**Supplementary Figure 5**. **Power-dependence TTA-upconversion of PdTPBP and perylene.** **a** TTA-upconversion intensity of PdTPBP (10 μM) and perylene (250 μM) dye pair in degassed toluene with different incident light power. **b** Double-logarithmic plot of integrated emission intensity as a function of 650 nm excitation power density was drawn. Solid lines illustrate a slope of 2.27 (black, quadratic) and a slope of 0.87 (red, linear), I_th_ is 88.5 mW cm^-2^.


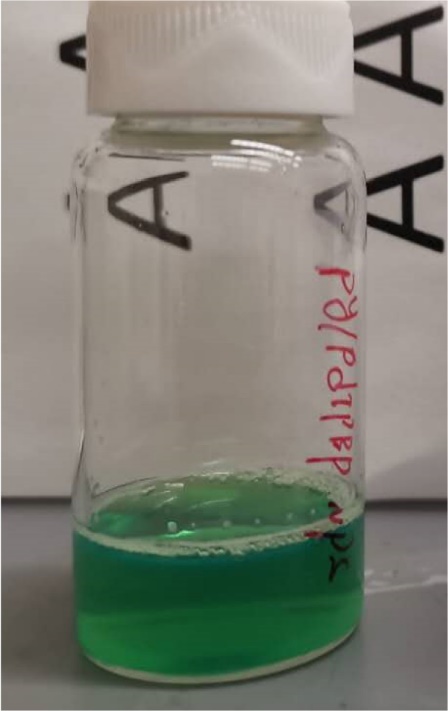


**Supplementary Figure 6**. A picture of the TTA-UCNP that we prepared.


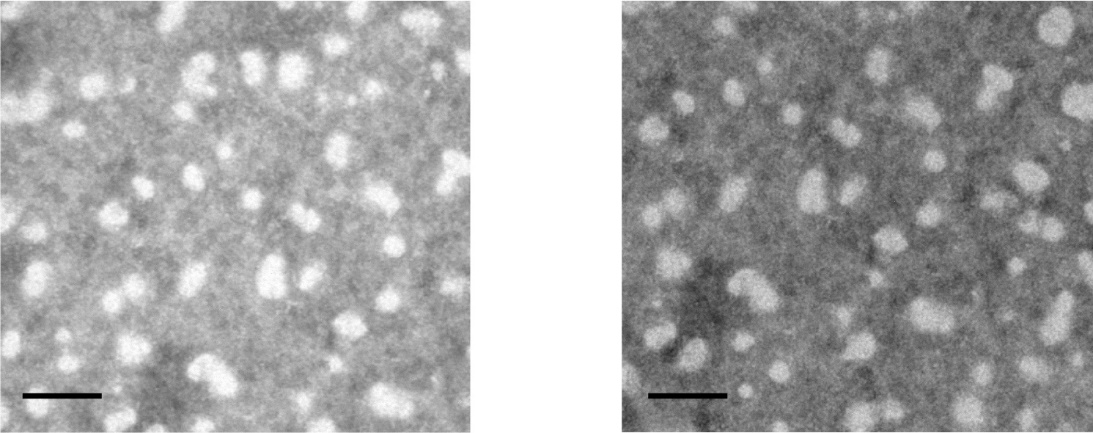


**Supplementary Figure 7**.  The transmission electron microscope image of TTA-UCNP after negative staining with sodium phosphotungstate. Black line represents scale bar which is 100 nm.

**
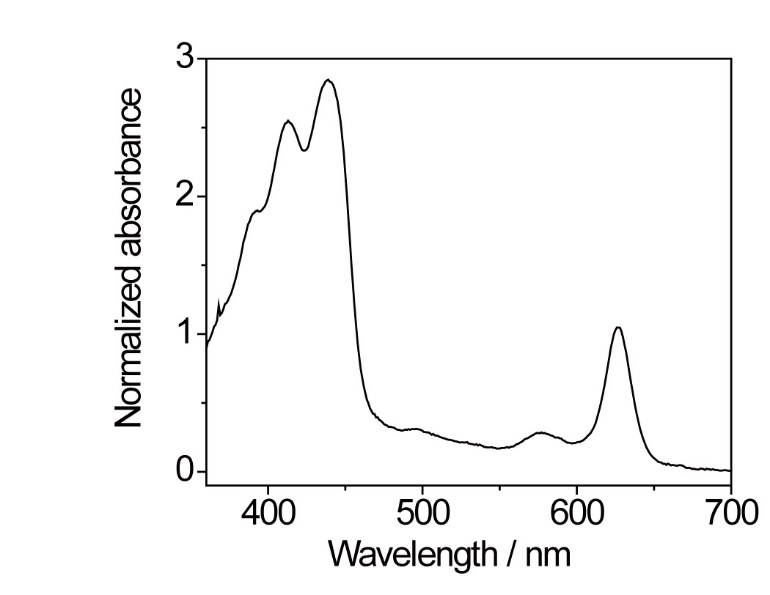
**

**Supplementary Figure 8**. Normalized UV-vis absorption spectra of TTA-UCNP in the PBS buffer.


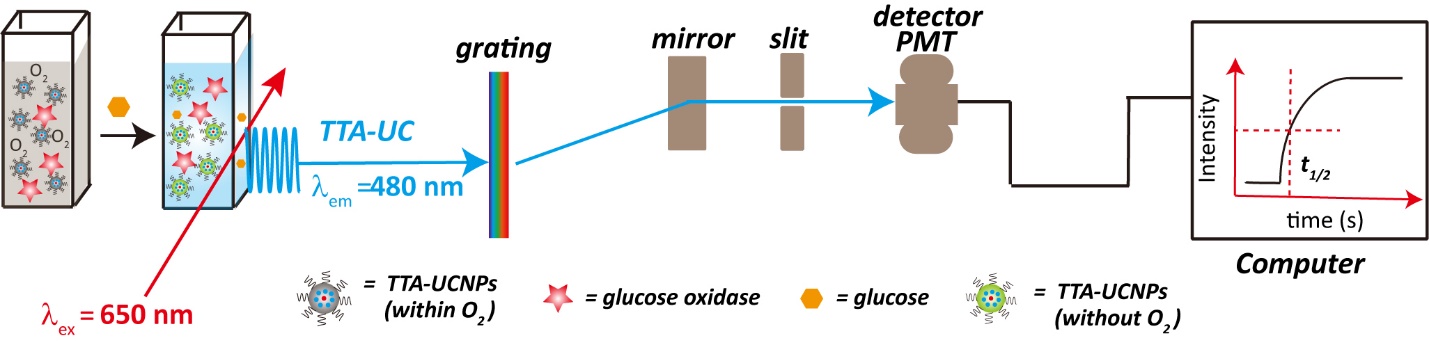


**Supplementary Figure 9**. The illustration of the process of TTA-UCNP in conjugation with GOX as the sensor to measure the glucose in aqueous solution.


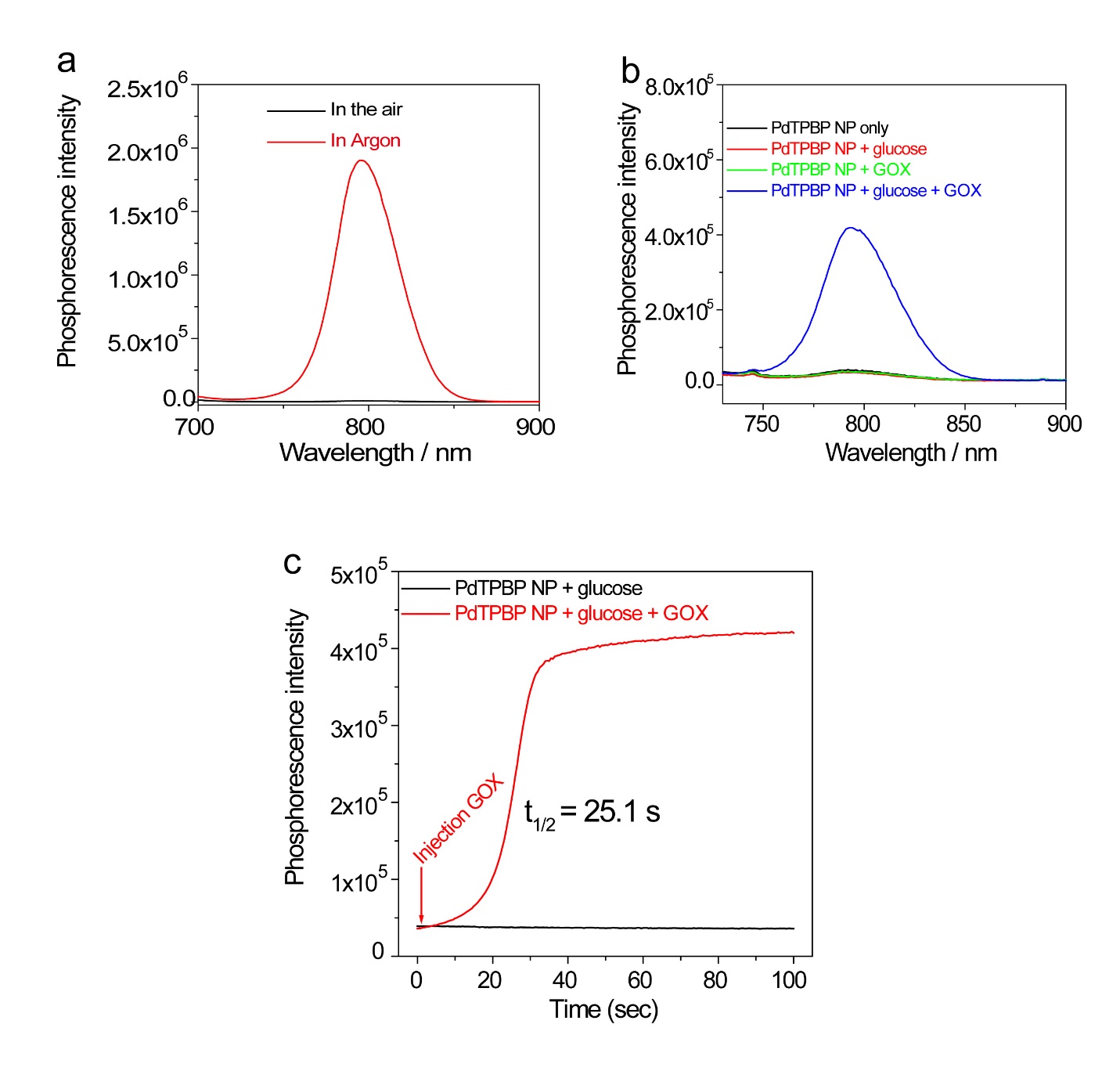


**Supplementary Figure 10**. **The phosphorescence intensity of PdTPBP under various conditions. a** The phosphorescence emission spectra of PdTPBP (10 µM) in toluene with air or argon. **b** Phosphorescence emission spectra of PdTPBP NP under different conditions (glucose or GOX alone, in the absence of glucose and GOX, and in the presence of both glucose and GOX) in PBS buffer. **c** The kinetic process of PdTPBP NP in the presence of GOX (15 µg mL^-1^) and glucose (5 mg mL^-1^). The half response time is 25.1 s, *λ*_ex_ = 650 nm.


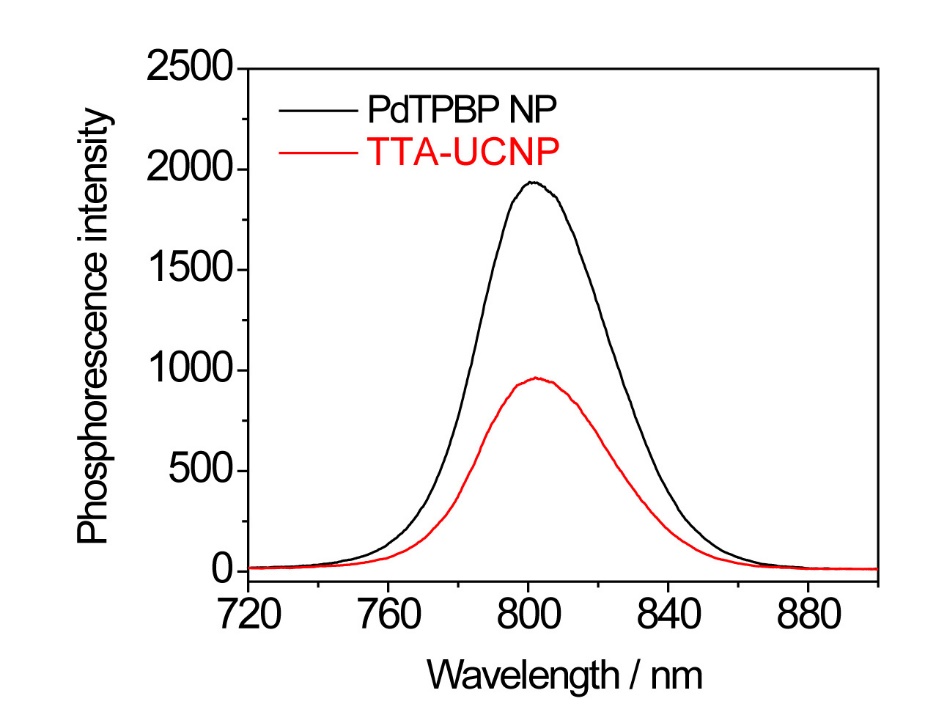


**Supplementary Figure 11**. Phosphorescence emission spectra of PdTPBP NP and the TTA-UCNP in the presence of glucose (5 mg mL^-1^) and GOX (37.5 µg mL^-1^). The phosphorescence emission spectra were recorded with a Hitachi F-7000 fluorescence spectrometer, *λ*_ex_ =630 nm.

.


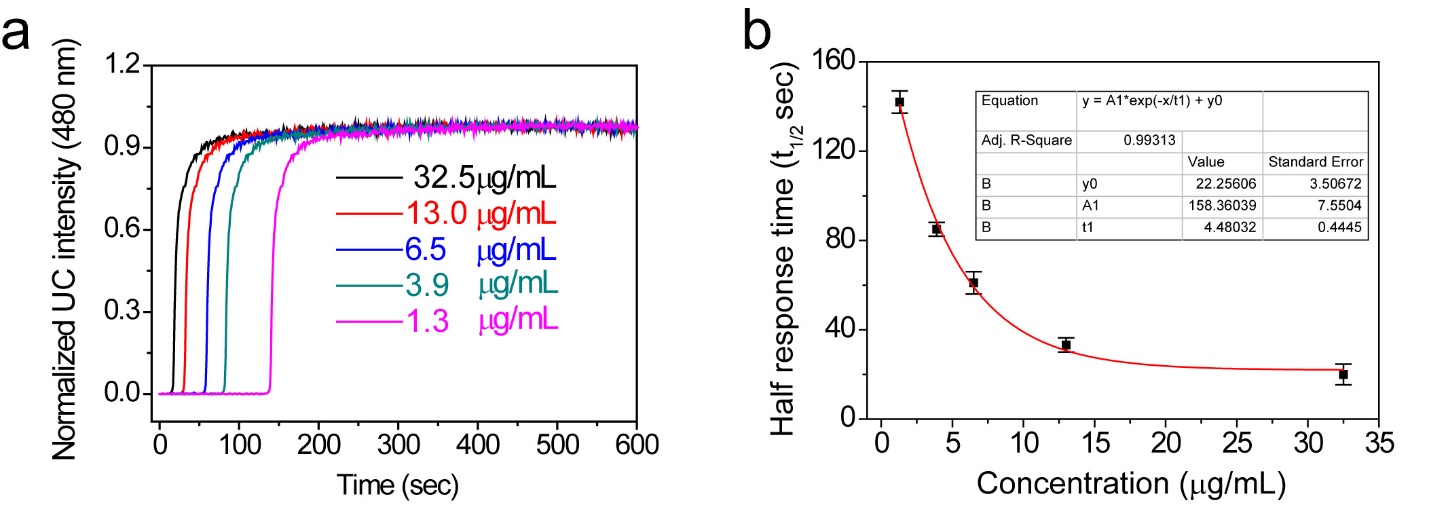


**Supplementary Figure 12. The impact of GOX concentration on the upconversion of TTA-UCNP. a** The representative kinetic process of the TTA-UCNP in the different concentration of GOX (1.3, 3.9, 6.5, 13.0, 32.5 µg mL^-1^) and glucose (5 mg mL^-1^). **b** The exponential relationship was found between the upconversion half response time and the concentration of GOX. *λ*_ex_ = 650 nm, 100 mW cm^-2^, n = 3 means that each experiment is repeated three times independently, the error bar represents the mean of the three times ± standard deviation (SD).


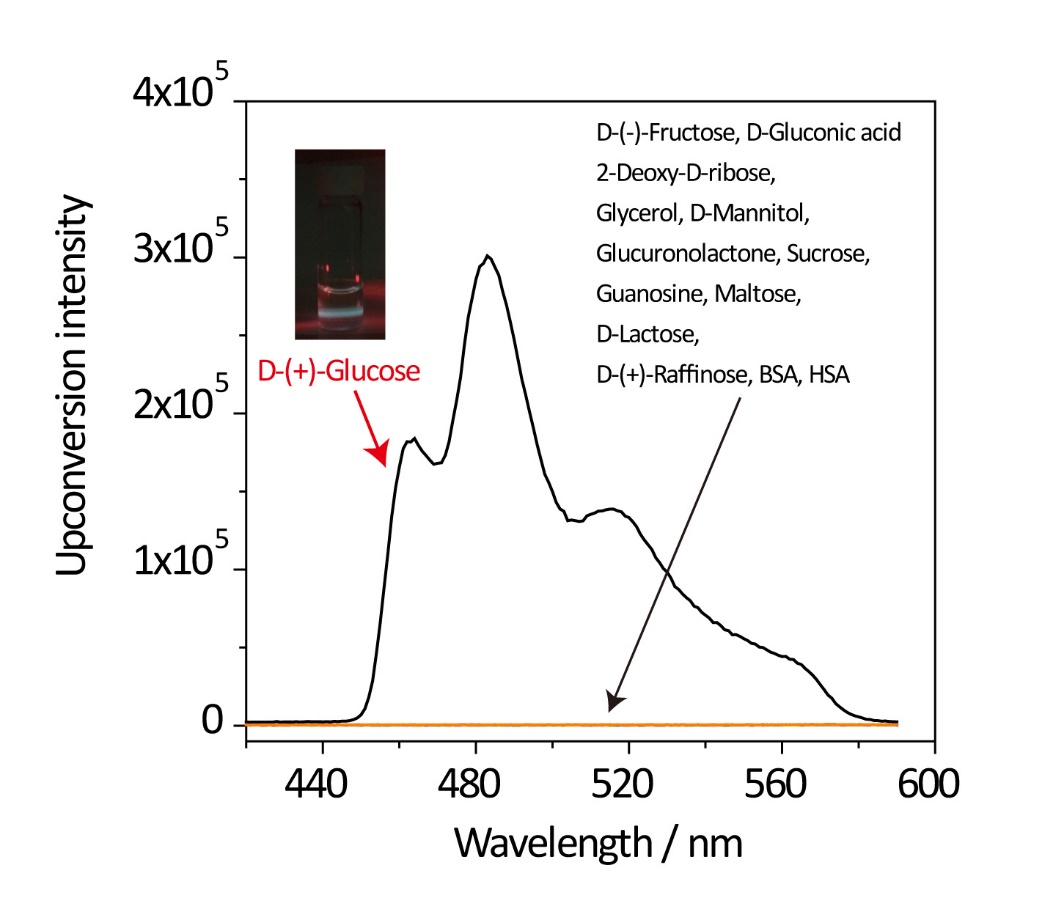


**Supplementary Figure 13.** The TTA-upconversion emission spectra of the mixture of TTA-UCNP and GOX (35.2 µg mL^-1^) in PBS buffer upon the addition of various analytes (0.7 mg mL^-1^) including D-(+)-glucose, D-(-)-Fructose, D-Gluconic acid, 2-D-dexy-D-ribose, Glycerol, D-Mannitol, Glucuronolactone, Sucrose, Guanosine, Maltose, D-Lactose, and D-(+)-Raffinose. *λ*_ex_ = 650 nm, 100 mW cm^-2^.


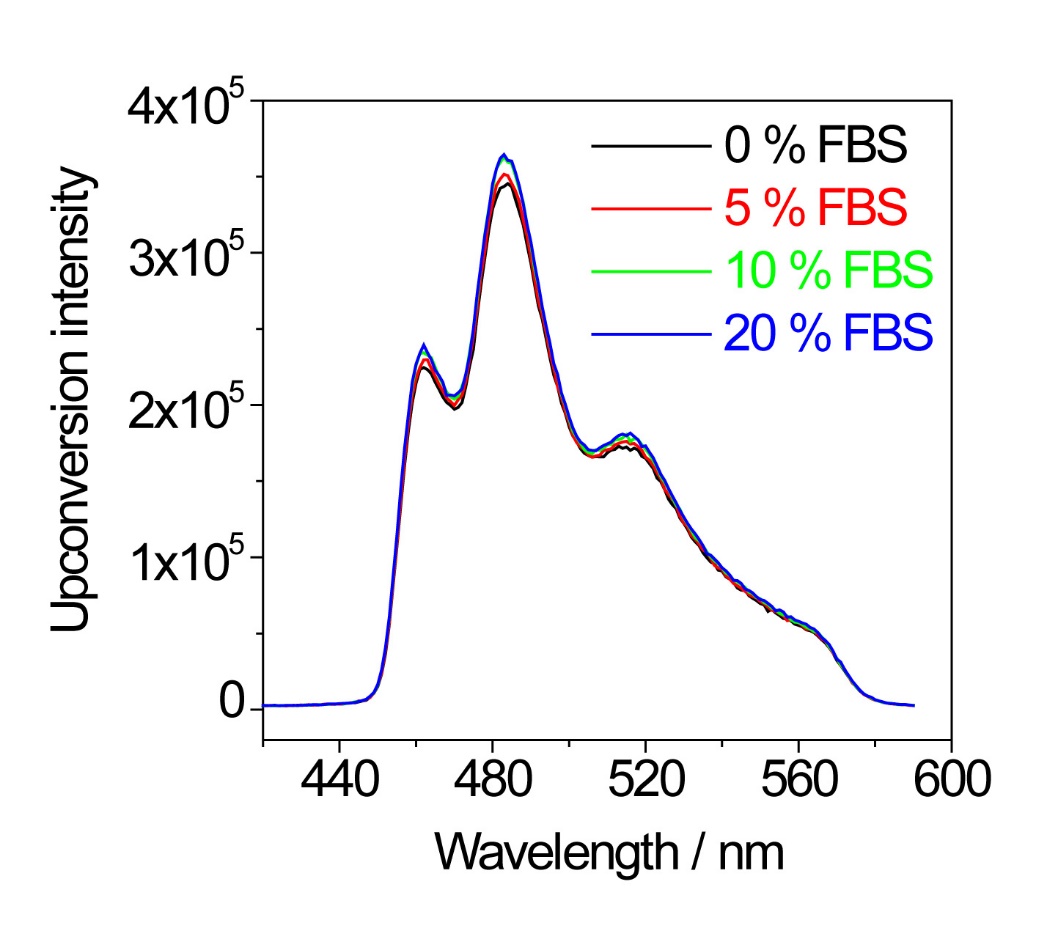


**Supplementary Figure 14**. TTA-upconversion spectra of TTA-UCNP in the presence of glucose (2 mg mL^-1^) and GOX (10 µg mL^-1^). *λ*_ex_ = 650 nm, 100 mW cm^-2^, in cell culture medium including 5%, 10%, 15% and 20% FBS.


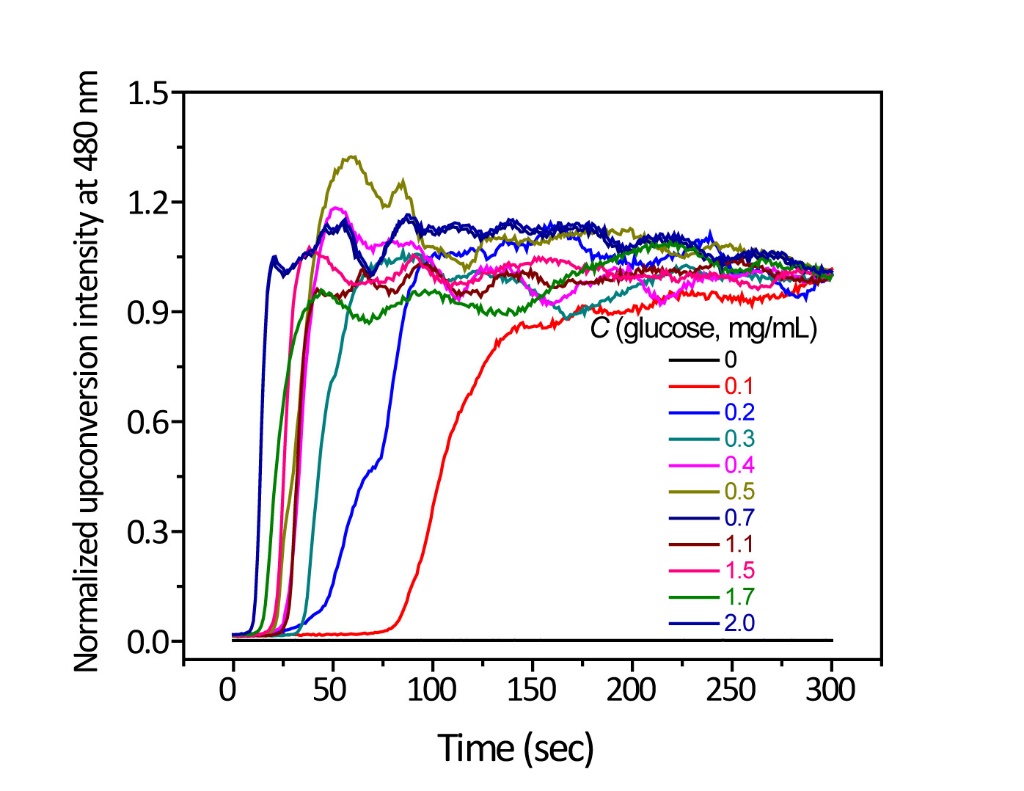


**Supplementary Figure 15**. The representative response times of the mixture of TTA-UCNP (1 mg mL^-1^) and GOX (10 µg mL^-1^) with different concentrations of glucose in the cell culture medium containing 20% FBS. *λ*_ex_ = 650 nm, 100 mW cm^-2^.


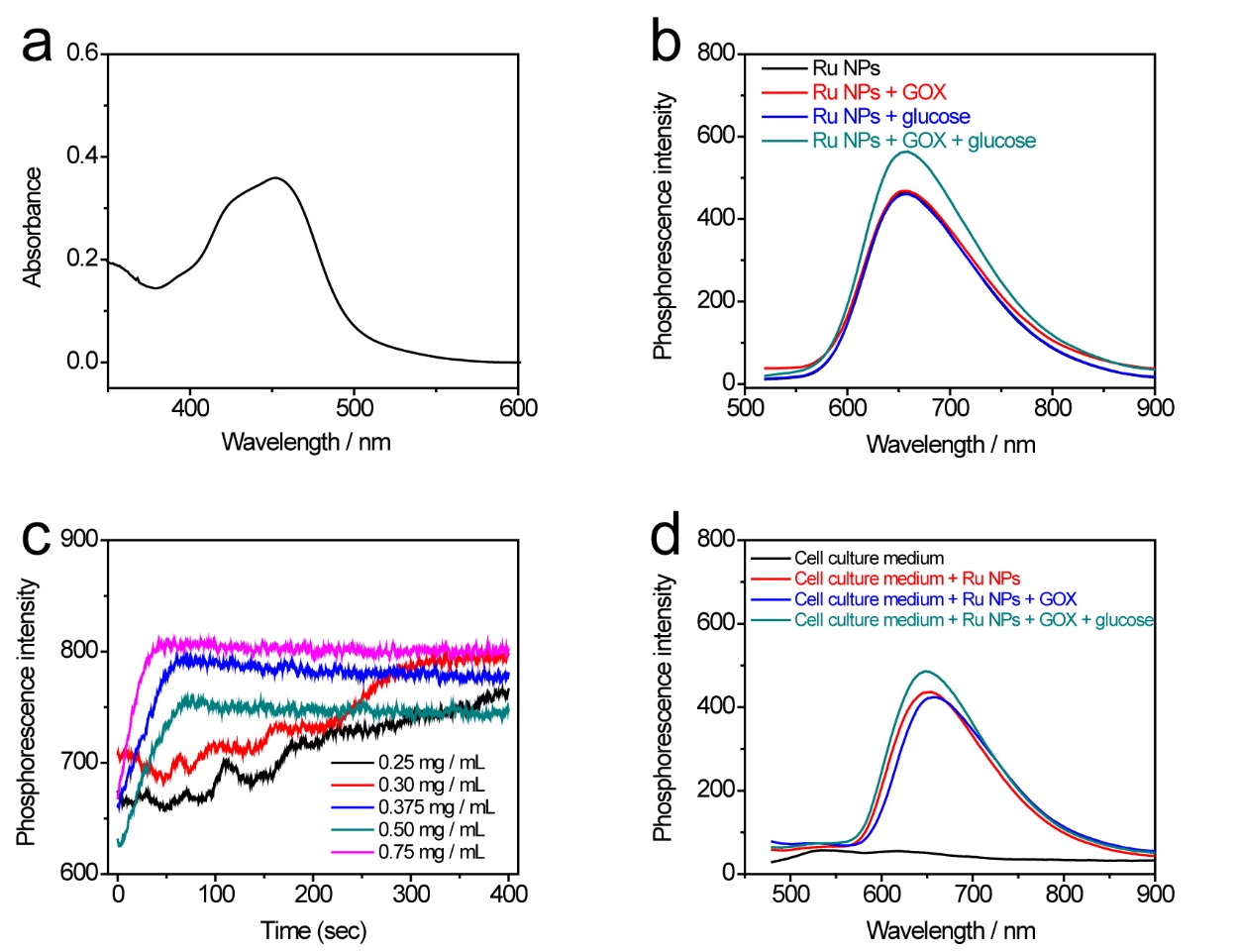


**Supplementary Figure 16**. **Ru NPs as the sensor for GOX sensing.** **a** UV-vis absorption spectrum of Ru NPs in deionized water. **b** Phosphorescence emission spectra of Ru NPs in different conditions in deionized water. GOX (10 µg mL^-1^), glucose (3.0 mg mL^-1^). **c** The response times of the mixture of Ru NPs and GOX (10 µg mL^-1^) in the different concentrations of glucose. *λ*_ex_ = 460 nm. **d** Phosphorescence emission spectra of Ru NPs in different conditions in the presence of cell culture medium. GOX (10 µg mL^-1^), glucose (3.0 mg mL^-1^). Ru NPs full name: Ru(bpy)_3_ nanoparticles.

**Supplementary Tables**

**Supplementary Table 1.** Typical examples to detect glucose in the literatures.

| Sensor | Excitation/Emission | AR: analytical range |
| --- | --- | --- |
| GOx and probe Ru(phen)^1^ | 488/610 nm | 0.7–10 mM |
| GOx and Ru(phen)^2^ | 468/570 nm | 0.5–15 mM |
| EuTC and Hypan^3^ | 400/616 nm | 0.1–2 mM |
| Anthracene based bis-PhBA^4^ | 370/423 nm | 0.3–1 mM |
| TTA-UCNP (this work) | 650/480 nm | 0.33-17 mM |

**Supplementary References**

1. Rosenzweig, Z.& Kopelman, R. Analytical Properties and Sensor Size Effects of a Micrometer-Sized Optical Fiber Glucose Biosensor. *Anal. Chem*. **68**, 1408-1413 (1996).

2. Bukowski, R. M. et al. Bright, Phase fluorometric glucose biosensor using oxygen as transducer and enzyme-doped xerogels, *Electron. Lett*. **43**, 202–204 (2007).

3. Schäferling. M, Wu. M, & Wolfbeis, O. S. Time-resolved fluorescent imaging of glucose, *J. Fluoresc*. **5**, 561–568 (2004).

4. James, T. D et al. Novel saccharide-photoinduced electron transfer sensors based on the interaction of boronic acid and amine, *J. Am. Chem. Soc*. **117**, 8982–8987 (1995).
