# Supplementary material for: Enzymatic enhancing of triplet–triplet annihilation upconversion by breaking oxygen quenching for background-free biological sensing
Source: Nat Commun. 2021 Mar 26;12:1898. doi: 10.1038/s41467-021-22282-1 (PMC7997900; doi:10.1038/s41467-021-22282-1)
Supplement: Supplementary file 3 — Reporting Summary [file 41467_2021_22282_MOESM3_ESM.pdf]

nature research

Corresponding author(s): Gang Han

Last updated by author(s): Dec 31, 2020

Reporting Summary

Nature Research wishes to improve the reproducibility of the work that we publish. This form provides structure for consistency and transparency in reporting. For further information on Nature Research policies, see our Editorial Policies and the Editorial Policy Checklist.

Statistics

For all statistical analyses, confirm that the following items are present in the figure legend, table legend, main text, or Methods section.

n/a	Confirmed	
☐	☒	The exact sample size (n) for each experimental group/condition, given as a discrete number and unit of measurement
☐	☒	A statement on whether measurements were taken from distinct samples or whether the same sample was measured repeatedly
☐	☒	The statistical test(s) used AND whether they are one- or two-sided<br>*Only common tests should be described solely by name; describe more complex techniques in the Methods section.*
☒	☐	A description of all covariates tested
☒	☐	A description of any assumptions or corrections, such as tests of normality and adjustment for multiple comparisons
☐	☒	A full description of the statistical parameters including central tendency (e.g. means) or other basic estimates (e.g. regression coefficient) AND variation (e.g. standard deviation) or associated estimates of uncertainty (e.g. confidence intervals)
☒	☐	For null hypothesis testing, the test statistic (e.g. F, t, r) with confidence intervals, effect sizes, degrees of freedom and P value noted<br>*Give P values as exact values whenever suitable.*
☒	☐	For Bayesian analysis, information on the choice of priors and Markov chain Monte Carlo settings
☒	☐	For hierarchical and complex designs, identification of the appropriate level for tests and full reporting of outcomes
☒	☐	Estimates of effect sizes (e.g. Cohen's d, Pearson's r), indicating how they were calculated

Our web collection on statistics for biologists contains articles on many of the points above.

Software and code

Policy information about availability of computer code

Data collection	Agilent Cary-5 spectrophotometer; Hitachi F-7000 fluorescence spectrometer; JEOL JEM-200CX transmission electron microscope (TEM); Malvern Zetasizer Nano ZS
Data analysis	UV-vis spectra were recorded via an Agilent Cary-5 spectrophotometer. Steady-state fluorescence spectra were measured with a Hitachi F-7000 fluorescence spectrometer. The morphology of the TTA-UC nanoparticle was characterized by using a JEOL JEM-200CX transmission electron microscope (TEM) operated at 80 kv. The sample for TEM measurement was prepared by dropping the solution onto a carbon-coated copper grid after negative staining with 10.0% (w/v) sodium phosphotungstic acid. The particle size and size distribution of the TTA-UCNP in aqueous solution was measured by using dynamic light scattering (DLS) via a Malvern Zetasizer Nano ZS. All statistical analyses were performed on Origin 8.5

For manuscripts utilizing custom algorithms or software that are central to the research but not yet described in published literature, software must be made available to editors and reviewers. We strongly encourage code deposition in a community repository (e.g. GitHub). See the Nature Research guidelines for submitting code & software for further information.

Data

Policy information about availability of data

All manuscripts must include a data availability statement. This statement should provide the following information, where applicable:

- Accession codes, unique identifiers, or web links for publicly available datasets
- A list of figures that have associated raw data
- A description of any restrictions on data availability

We have provided a full data availability statement in the manuscript.

Field-specific reporting

Please select the one below that is the best fit for your research. If you are not sure, read the appropriate sections before making your selection.

- [x] Life sciences
- [ ] Behavioural & social sciences
- [ ] Ecological, evolutionary & environmental sciences

For a reference copy of the document with all sections, see nature.com/documents/nr-reporting-summary-flat.pdf

Life sciences study design

All studies must disclose on these points even when the disclosure is negative.

Sample size	The size of the sample is based on previous experimental experience, a total of about 200 mL of nanoparticles are used
Data exclusions	No data exclusion
Replication	The experiments was repeated, and the results of the experiment were reproducible
Randomization	The nanoparticles were randomly assigned to each group
Blinding	blinding was applied to the substrate selectivity experiment

Reporting for specific materials, systems and methods

We require information from authors about some types of materials, experimental systems and methods used in many studies. Here, indicate whether each material, system or method listed is relevant to your study. If you are not sure if a list item applies to your research, read the appropriate section before selecting a response.

Materials & experimental systems

n/a	Involved in the study	
x		Antibodies
x		Eukaryotic cell lines
x		Palaeontology and archaeology
x		Animals and other organisms
x		Human research participants
x		Clinical data
x		Dual use research of concern

Methods

n/a	Involved in the study	
x		ChIP-seq
x		Flow cytometry
x		MRI-based neuroimaging
